# Olfactory Responses of *Apis mellifera* and *Bombus terrestris* to Floral Volatiles from Three Solanaceae Crops

**DOI:** 10.3390/insects17050507

**Published:** 2026-05-15

**Authors:** Yingying Sun, Jisu Jin, Guangyuan Jiao, Xiaolei Huang, Chao Chen, Hong Zhang

**Affiliations:** State Key Laboratory of Resource Insects, Institute of Apicultural Research, Chinese Academy of Agricultural Sciences, Beijing 100193, China; sunyingying2114@163.com (Y.S.); jinjisu9110@163.com (J.J.); jiaoguangyuan0206@163.com (G.J.); 13327415540@163.com (X.H.)

**Keywords:** *Apis mellifera*, *Bombus terrestris*, floral volatiles, EAG, behavioral response

## Abstract

The efficiency with which solanaceous crops are pollinated is determined by pollinator behavioral preferences. Although *Bombus terrestris* typically outperforms *Apis mellifera* in this respect, the chemo-ecological mechanisms underlying their divergent olfactory responses remain insufficiently determined. We combined behavioral assays, gas chromatography–mass spectrometry, and electroantennogram recordings to compare the responses of these bee species to the volatile organic compounds (VOCs) emitted by tomato, pepper, and eggplant flowers. Whereas *B. terrestris* showed a strong foraging preference for all three crop plants, *A. mellifera* displayed distinct avoidance. Chemical analyses identified 82, 63, and 60 VOCs in tomato, pepper, and eggplant flowers, respectively. Among the 14 VOCs commonly emitted by all three crops, linalool, nerol, (*E*,*E*)-2,4-decadienal, 2-hexenal, tridecanal, and (*E*,*Z*)-2,6-nonadienal elicited significantly different electrophysiological responses in the two bee species, and are, thus, considered key compounds mediating their behavioral differences. Moreover, in behavioral assays, *A. mellifera* and *B. terrestris* showed significantly different responses to four concentration levels of linalool, nerol, (*E*,*E*)-2,4-decadienal, and tridecanal. This study elucidates the plant–pollinator olfactory interactions that contribute to determining the different foraging behaviors of two bee species in pollinating solanaceous crops, thereby providing a theoretical basis for optimizing pollinator attraction strategies.

## Simple Summary

The yields of solanaceous crops, including tomato (*Solanum lycopersicum*), pepper (*Capsicum annuum*), and eggplant (*Solanum melongena*), depend particularly on the pollination services provided by insects. However, whereas bumblebees (*Bombus terrestris*) are highly efficient pollinators of these crop plants, honeybees (*Apis mellifera*) frequently avoid their flowers. The mechanisms associated with this behavioral divergence have yet to be sufficiently clarified, and given the potential commercial implications, in this study, we investigated the olfactory perception and behavioral responses of these two bee species to volatiles emitted by the flowers of tomato, pepper, and eggplant. Compared with *A. mellifera*, *B. terrestris* displayed significantly higher olfactory sensitivity to specific floral volatiles, whereas these compounds often failed to elicit preferential response in honeybees. Our findings thus enabled us to characterize the olfactory interactions between plants and bees mediated by floral volatiles, and will provide a scientific basis for developing novel strategies to attract pollinators in agricultural ecosystems, thereby enhancing crop pollination efficiency and promoting sustainable and eco-friendly agricultural production.

## 1. Introduction

Bees, of which there are approximately 25,000 known species worldwide, including honeybees, bumblebees, solitary bees, parasitic bees, and stingless bees, play pivotal ecological roles as pollinators of plants [1,2,3]. An estimated 85% of the world’s major crops are pollinated by bees and other insects [4,5], with greenhouse vegetables and fruits being particularly dependent on the pollinating services of bees [6]. Bee pollination enhances plant fertilization and fruit set, subsequently increasing both yields and size of the fruits, as well as enhancing flavor and nutrient content [7,8,9,10]. In addition, whilst foraging for pollen and nectar, pollinating bees disperse pollen over potentially considerable distances, thereby contributing to the expansion of plant distribution ranges and increasing the genetic diversity within populations. As a natural pollinator, the services of bees can reduce the dependence on chemical pesticides and artificial pollination, thereby mitigating the negative environmental impacts of agricultural production and, thus, highlighting the indispensable roles played by bees in promoting sustainable agriculture and maintenance of an ecological balance [11].

The Solanaceae family, comprising 150 genera and approximately 2500–3000 species, is among the most commercially important angiosperm families worldwide, ranking third in terms of economic value, and is paramount among the nutritionally important vegetable groups [12,13], with crops such as tomatoes, peppers, and eggplants in high demand for human consumption. Current methods adopted for the pollination of solanaceous plants include artificial, hormone-assisted, and bee pollination [14,15]. Whereas animal pollination is globally essential, it is of particular significance within the Solanaceae family, on account of the specialized floral morphologies of these plants, such as poricidal anthers. Under conditions of sub-optimal pollination, there are often substantial yield deficits in key crops, including tomatoes and eggplants. For example, inadequate pollinator visitation can result in a 15–40% reduction in fruit weight and declines in seed set exceeding 50% [16]. Given this high degree of reproductive dependency, characterizing the specific chemical cues that mediate pollinator attraction is essential for mitigating such yield losses and ensuring agricultural stability in solanaceous cropping systems. Among the diverse range of pollinating insects, bees have been established to have the highest efficiency, surpassing the contributions of other species in terms of both efficacy and outcome, making these insects the preferred agents for maximizing crop production. Honeybees (*Apis mellifera*) and bumblebees (*Bombus terrestris*) are the most widely used pollinators of solanaceous crops. However, given that *A*. *mellifera* is highly sensitive to floral scents and is more attracted to nectar with a high sugar content, tomato plants, the flowers of which lack nectar, are less appealing [17]. In contrast, the abundant pollen rewards provided by these crops act as a strong attractant to *B*. *terrestris*. In general, the efficiency with which *A. mellifera* pollinate solanaceous crops is relatively poor, as these bees experience difficulties in effectively dislodging pollen, leading to reduced rates of fruit set and a higher percentage of deformed fruit. Given their more rapid rates of flower visits and superior pollination efficiency [18,19]. *B. terrestris* is more commonly used to pollinate solanaceous crops. Notably however, to date there have been comparatively few studies that have sought to examine the response of *A. mellifera* and *B. terrestris* to the floral volatiles emitted by these crops, thereby highlighting our currently poor understanding of the pollinator preferences and efficiencies of these bees at the molecular level [20,21].

Plants deploy an array of cues and signals to attract and modify the behavior of pollinators, including flower color, shape, and scent [22,23,24,25]. With respect to scent, pollinating bees are predominantly dependent on olfaction for the detection of floral odors [26], which facilitates an assessment of pollen quality. Consequently, floral volatiles serve as essential cues enabling pollinators to locate and identify flowers [27]. These volatiles primarily comprise monoterpenes, diterpenes, phenols, simple ketones, alcohols, and esters [28,29,30], and floral odors often contain multiple chemical components, with one or more at high concentrations. Moreover, it has been established that compounds such as linalool and acetophenone not only attract pollinators but also parasitoid wasps seeking prey, thereby enhancing the efficacy of natural enemies in agricultural ecosystems and thus serving as an effective means of biological control [31]. Plants differ considerably regarding the profiles of volatiles emitted by their flowers, and *A. mellifera* and *B. terrestris* have been found to show distinct preferences for these volatiles, with different pollinating bees responding uniquely to floral odors. For example, *B. terrestris* shows a preference for β-pinene released by broad-leaved groundsel, which acts as an attractant for these pollinators [32]. In contrast, the scent profile of apple blossom consists predominantly of aromatic compounds supplemented by aliphatic and terpenoid compounds, among which benzaldehyde, the most abundant aromatic compound, has been established to have a significant effect on *A. mellifera* [33]. Similarly, there is evidence to indicate that *A. mellifera* and *B. terrestris* show differing responses to volatiles emitted by the flowers of fruit crops, such as kiwifruit [34], pears, melons, and strawberries [35], thereby highlighting the complexity of pollinator–flower interactions.

In this study, we sought to determine differences in the olfactory response*s* of *A. mellifera* and *B. terrestris* to volatiles emitted by the flowers of three types of solanaceous crop plants. By integrating methods from behavioral science and analytical chemistry, we specifically analyzed the distinct floral visitation behaviors of these two species, with a particular focus on identifying the specific chemicals responsible for the differing foraging behaviors of the two bee groups. By adopting a chemo-ecological perspective, with a view toward gaining an understanding of disparities in the performance of *A. mellifera* and *B. terrestris* in pollinating solanaceous crops, this study addresses issues such as low visitation rates and inefficient pollination by pollinators in certain crops. Our findings will contribute to broadening application scenarios for both *A. mellifera* and *B. terrestris*, and thereby the development of sustainable agricultural ecosystems.

## 2. Materials and Methods

### 2.1. Plants and Bees

The three solanaceous crop species used in this study, tomato (*Solanum lycopersicum*), pepper (*Capsicum annuum*), and eggplant (*Solanum melongena*), of which the respective varieties were ‘Shouhe Fenguan’, ‘Wangniu’, and ‘Lvbao No. 1’, were purchased from the Shouhe Seed Industry Store in Taobao. Plant were cultivated in a greenhouse at the Institute of Apicultural Research, Chinese Academy of Agricultural Sciences, Beijing, China, in an environment with a temperature of 26 ± 1 °C and relative humidity of 65 ± 5%, under a light–dark cycle of 16 h (day)/8 h (night). To eliminate the effects of abiotic factors, all plants were grown in the same environment.

To ensure biological independence and experimental reliability, foragers were randomly collected from three independent and healthy colonies located at the Institute of Apicultural Research, Chinese Academy of Agricultural Sciences. All experiments were conducted using female worker bees (*A. mellifera* and *B. terrestris*), captured only when they were observed actively collecting nectar or pollen. Although the exact age in days was not tracked, this behavioral criterion enabled us to ensure a consistent physiological state associated with foraging. All source colonies were queenright and healthy and regularly inspected for pathogens to assess the vigor of the experimental subjects. All bees were housed in incubators (Daskate Biotechnology Co., Ltd., Hefei, China) at 30 °C and 60% relative humidity [36,37].To simulate natural diurnal patterns, *A. mellifera* were maintained under a 16 h/8 h light–dark cycle. In contrast, buff-tailed *B. terrestris* were maintained under conditions of continuous darkness (24 h dark). Although CO_2_ levels were not continuously monitored, the greenhouse was equipped with an automated ventilation system, thereby facilitating a constant exchange of air with the outside environment. This contributed to minimizing potential CO_2_ fluctuations that might otherwise have influenced insect respiration. Throughout the experiment, for sustenance, the bees were provided with a 50% (*w*/*w*) sucrose solution, prepared by dissolving 400 g analytical-grade sucrose (Beijing Solarbio Science & Technology Co., Ltd., Beijing, China) in 400 g distilled water. The mixture was stirred at room temperature until completely dissolved, after which the solution was sterilized by autoclaving at 121 °C for 15 min and thereafter stored at 4 °C. To prevent fermentation, fresh solutions were prepared at 12 h intervals.

### 2.2. Olfactory Selection Behavior

For the purposes of olfactory choice experiments, we used Y-tube olfactometers, focusing on the behavioral preferences of *A. mellifera* and *B. terrestris* toward flowers of the assessed crop plants and selected test volatiles. These experiments were conducted in a climate-controlled laboratory, in which environmental conditions were maintained at a constant temperature of 25 ± 1 °C and relative humidity of 60 ± 5%, which were monitored throughout the experiment to ensure consistency and minimize environmental-induced variance in insect behavior. The olfactometers (Zhengzhou Dongke Glass Factory, Zhengzhou, China), constructed from high-borosilicate glass, comprised a 20 cm main arm and two 15 cm side arms projecting at an angle of 60°, with an inner diameter of 4 cm [38]. Air was drawn through an atmospheric sampler (QC-3; Beijing Institute of Labor Instrument, Beijing, China) and filtered through an activated carbon filter and a distilled water humidifier bottle to produce a clean, humidified airflow [39,40]. The rate at which air was flowed was maintained at 500 mL/min using a glass rotameter (LZB-3WB; Xiangjin Flow Meter Factory, Jinhu, China) and used to convey the test volatiles from the odor source vial into the Y-shaped olfactory apparatus used for bee selection. All components were connected via flexible tubing. To minimize external visual cues, experiments were conducted under red-light conditions, and to standardize the motivation levels of the selected foraging bees, these were starved for 4 h prior to trials [41].

During each odor-selection test, a single bee was introduced at the entrance of the main arm of the apparatus. Observations were continued for 5 min, during which a selection was considered valid if the bee entered a branch arm beyond the two-thirds mark and remained therein for at least 5 s. If the bee did not show any preference for an odor within the allotted time, the test was considered invalid. Each bee was used for only a single trial. The study was performed using eight groups of bees, each comprising 10 individuals. To minimize positional bias, the side-arm positions were alternated after five consecutive trials, and a different Y-tube olfactometer with fresh test odors was used for each group. Glassware, including the Y-tube olfactometers, was cleaned using anhydrous ethanol and distilled water, then baked at 80 °C for 3 h to ensure sterility and prevent contamination.

For assessments of the preferential responses toward crop plant floral odors, an odor source bottle containing two fresh full-bloom flowers and an empty odor source bottle were received by the experimental and control groups, respectively. To assess the preferential selection of volatile organic compounds (VOCs) for the experimental group bees, we used filter paper (1 cm × 4 cm) impregnated with 10 µL of the test compound, whereas filter paper to which 10 µL of hexane had been added was used for the control group bees. Having initially allowed the test and control compounds to evaporate for 30 s prior to testing, we recorded the number of preferences shown by *A. mellifera* and *B. terrestris* toward the three types of flowers and the different volatiles.

Following data collection, rates of attraction were calculated using the following formula: Attraction (%) = (number of bees in the experimental group/total number of bees tested) × 100%.

### 2.3. Extraction and Identification of Floral Volatiles

Samples (0.6 g) of fresh flowers were ground cryogenically in liquid nitrogen, with the resulting material being placed in 20 mL amber sample vials (Thermo Fisher Scientific, Waltham, MA, USA), to which was added 6 mL of saturated NaCl solution (Beijing Solarbio Science & Technology Co., Ltd., Beijing, China) [42], with 6 mL of saturated NaCl solution serving as a control. Samples were placed in a 37 °C water bath for 50 min. The extraction needle (65 μm polydimethylsiloxane/divinylbenzene, Supelco Analytical, Bellefonte, PA, USA) was inserted into the brown vial to a depth of 1–2 mm below the liquid surface. Adsorption occurred at 37 °C and 600 rpm for 30 min. The resulting extract was used for gas chromatography–mass spectrometry (GC-MS) analysis. In this regard, it should be noted that while we characterized the relative proportions of floral volatiles, absolute rates of emission were not determined. Accordingly, further studies are warranted in which the use of internal standards would provide a more precise ecological context regarding the total scent output perceived by pollinators.

Samples of the extracts obtained from the flowers of solanaceous plants were analyzed using a GC-MS system (GC-MS 2010 plus; Shimadzu, Tokyo, Japan), equipped with a DB-5MS capillary column (30 m × 0.25 mm × 0.25 µm, Sigma-Aldrich, St. Louis, MO, USA). The carrier gas was high-purity helium (purity ≥ 99.999%), flowing at a constant rate of 1 mL/min. For volatile analysis, a solid-phase microextraction (SPME) fiber was desorbed in the GC splitless injection port at 250 °C for 5 min. The GC conditions were as follows: an initial temperature of 40 °C for 5 min, followed by a 6 °C/min ramp to 130 °C, then a 10 °C/min ramp to 250 °C, at which it was held for 5 min. MS conditions were as follows: an ion source temperature of 250 °C, interface temperature of 250 °C, scan mass range of 33–500 m/z, and ionization mode electron impact of 70 eV. Each sample was assessed in triplicate, with the data obtained being analyzed using GCMS Version 4.30 software (Shimadzu, Kyoto, Japan). Analysis of odor volatile components was based on reference to the NIST 20 mass spectrometry library. On the basis of the total ion chromatograms of the volatiles obtained from tomato, eggplant, and pepper, compounds with a similarity index of >85 and stable detection in at least two replicates were selected for subsequent analyses.

Values for the single-component percentage were calculated using the following formula: Single-component percentage (%) = (peak area of a single component/total area of volatile components) × 100%.

### 2.4. Electroantennography (EAG)

To identify chemical substances that effectively stimulate the antennae of *A. mellifera* and *B. terrestris*, we performed electroantennography (EAG). The test bees were placed beneath a stereomicroscope and gently restrained using forceps to ensure minimal stress. The right antennae were excised using a sharp scalpel, followed by careful trimming of the base and flagellar tips. The remaining segments were immersed in a glass capillary filled with physiological saline solution. During this procedure, meticulous care was taken to prevent the formation of air bubbles. The antenna base was connected to a reference electrode, whereas its tip was attached to a recording electrode, both of which were interfaced with a DC/AC amplifier [39] (IDAC-2; Syntech, Kirchzarten, Germany). The amplified signal was transmitted to a computer via an output port, enabling real-time acquisition and analysis of the experimental data. This setup ensured accurate and reliable EAG readings, which are essential for deciphering the antennal responses to chemical stimuli. Only preparations for which we obtained stable baselines and robust responses to the positive control were used in the final analysis.

The selected compounds were used as odor stimulants in the EAG experiments. For each trial, 10 μL of the sample was applied to a 1 cm × 4 cm filter paper, which was folded into a V-shape and inserted into a 1 mL pipette tip to constitute the experimental group. Solvents (n-hexane or ethanol) served as negative controls, whereas 1 μg/μL citral [43] was used as a positive control. A stimulus gas flow generator (CS-55; Syntech, Kirchzarten, Germany) delivered humidified gas at a rate of 300 mL/min [44]. Each stimulus was administered for 0.5 s, with a 30 s interval between successive stimuli. For each of the assessed antennae, stimuli were applied in the sequence n-hexane and citral, followed by increasing concentrations (0.1, 1, 10, and 100 μg/μL) of the test compound, and concluded with 1 μg/μL citral and n-hexane. For each concentration of the test compounds, we used six antennae as replicates to ensure data reliability, and for each repetition, a fresh antenna was used to maintain consistency. EAG data were recorded using EAG Pro (V2.1.0) software (Syntech) to facilitate precise monitoring and analysis of antennal responses. The concentrations of the assessed compounds were selected based on literature-reported rates of Solanaceae floral volatile emissions and the results of preliminary pilot studies conducted to ensure they were within the active sensory range for the target pollinators. Although not representing a comprehensive ecological range, these values nevertheless enabled us to evaluate the physiological and behavioral thresholds of both *A. mellifera* and *B. terrestris* under comparable conditions [45].

Values of the relative responses to the assessed compounds were calculated using the following formula: Relative response value = [(average value of the compound being tested − average value of the blank control)/(average value of the positive control − average value of the blank control)] × 100%.

### 2.5. Statistical Analysis

The significance of behavioral differences was assessed using the chi-square test, reporting both the *χ*^2^ value and the corresponding *p*-value. Relative EAG response values were analyzed using an independent-samples *t*-test [42]. Statistical significance was assessed at the *p* < 0.05 and *p* < 0.01 levels. To account for the multiple comparisons involved in testing multiple concentrations, *p*-values were adjusted using the Benjamini–Hochberg false discovery rate procedure with a significance threshold of *Q* = 0.05. Effect sizes were quantified using Cohen’s *d* metric, calculated as the difference between the means of the two species divided by the pooled standard deviation. The magnitude of the effect was interpreted as small (0.2 < *d* < 0.5), medium (0.5 < *d* < 0.8), or large (*d* > 0.8). Graphical presentation of the data was facilitated using GraphPad Prism version 9.5.1 (GraphPad Software, Boston, MA, USA), and data analysis was conducted using SPSS version 25.0 (IBM, Armonk, NY, USA).

## 3. Results

### 3.1. Comparisons of the Behavioral Preferences of A. mellifera and B. terrestris for the Flowers of Three Solanaceous Crop Plants

**Figure 1:**
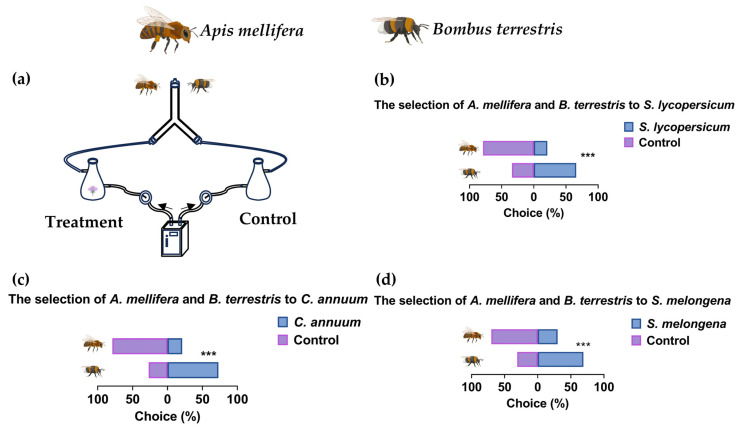
Behavioral responses of *Apis mellifera* (*A. mellifera*) and *Bombus terrestris* (*B. terrestris*) to the floral odors of three solanaceous crop plants. (**a**) Schematic diagram of the Y-tube olfactometer illustrating the direction of airflow, with the treatment arm containing fresh flowers and the control arm being empty, the arrows represent the direction of airflow. (**b**) Differential olfactory preferences of *A. mellifera* and *B. terrestris* for tomato floral odors (*χ*^2^ = 31.02, *df* = 1, *p* < 0.001, *n* = 80). (**c**) Differential olfactory preferences for pepper floral odors (*χ*^2^ = 42.19, *df* = 1, *p* < 0.001, *n* = 80). (**d**) Differential olfactory preferences for eggplant floral odors (*χ*^2^ = 24.03, *df* = 1, *p* < 0.001, *n* = 80). (***: *p* < 0.001).

The test results were as follows: 53/80 (66%) *B. terrestris* and 18/80 (22%) *A. mellifera* selected tomato flowers; 58/80 (73%) *B. terrestris* and 17/80 (21%) *A. mellifera* selected pepper flowers; and 55/80 (69%) *B. terrestris* and 24/80 (30%) *A. mellifera* selected eggplant flowers. The preference difference between *A. mellifera* and *B. terrestris* for tomato flower odors was significant (*χ*^2^ = 31.02, *df* = 1, *p* < 0.001, *n* = 80). The preference difference between *A. mellifera* and *B. terrestris* for pepper flower odors was significant (*χ*^2^ = 42.19, *df* = 1, *p* < 0.001, *n* = 80). The choice difference between *A. mellifera* and *B. terrestris* for eggplant flower odors was significant (*χ*^2^ = 24.03, *df* = 1, *p* < 0.001, *n* = 80). These results indicate that *A. mellifera* and *B. terrestris* differ significantly in their preference for odors emitted from the three Solanaceae flower. Specifically, whereas *B. terrestris* showed a marked attraction, *A. mellifera* exhibited a lack of preference for any three flower types (Figure 1).

### 3.2. Identification of Common Volatile Compounds in Flowers of the Three Solanaceous Crop Plants

**Figure 2:**
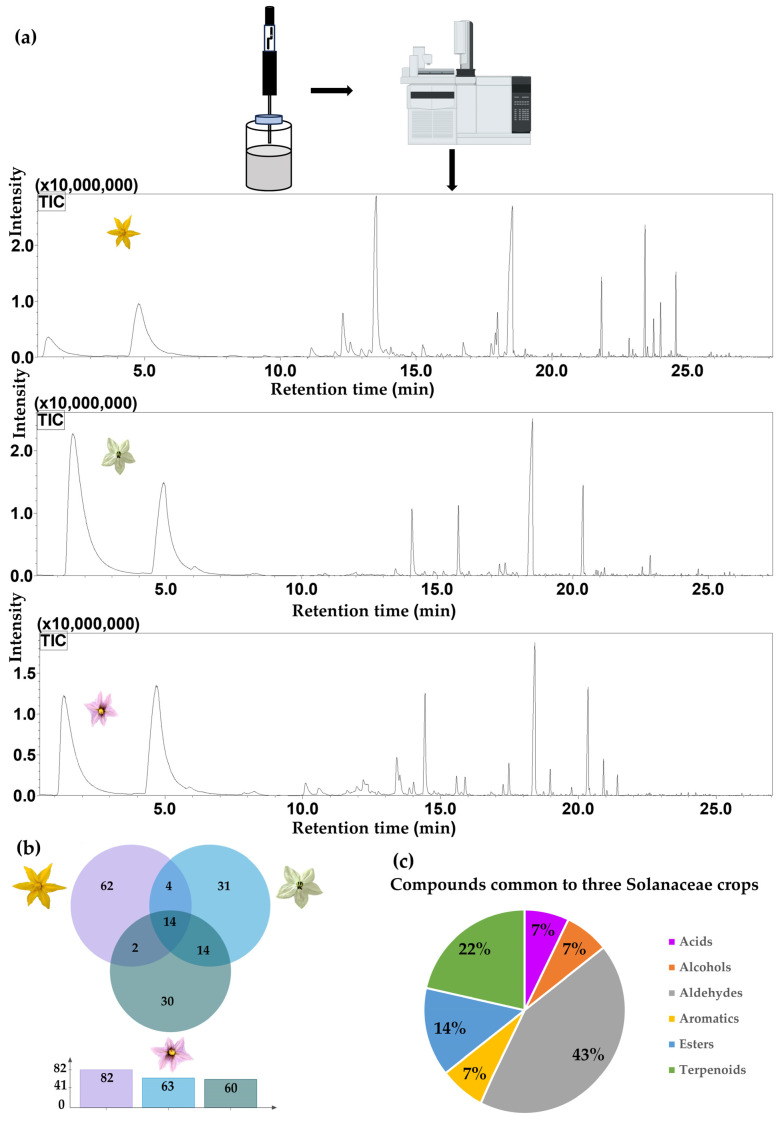
Analysis of floral volatile organic compounds (VOCs) from three solanaceous crop plants. (**a**) Schematic representation of the solid-phase microextraction procedure (**top**), the gas chromatography–mass spectrometry analytical workflow (**middle**), and the total ion chromatograms of floral volatiles from tomato, pepper, and eggplant (**bottom**). (**b**) Venn diagram illustrating the intersection of common and unique VOCs among the three solanaceous crop plants. (**c**) Chemical classification and relative proportions of the 14 common VOCs identified in all three species.

The major classes of VOCs include acids, alcohols, aldehydes, alkanes, amines, aromatics, esters, ketones, and olefins. We detected 82 VOCs in tomato flowers, 63 in pepper flowers, and 60 in eggplant flowers (Supplementary Table S1), among which, tomato flowers were characterized by 62 unique VOCs, pepper flowers had 31 unique compounds, and eggplant flowers contained 30 unique compounds. Tomato and pepper flowers had 18 compounds in common, whereas tomato and eggplant flowers had 16 in common, and 28 were common to both the pepper and eggplant flowers. Moreover, the following 14 VOCs were produced by the flowers of all three crop plants: (*E*,*E*)-2,4-decadienal, (*E*,*E*)-2,4-hexadienal, (*E*,*Z*)-2,6-nonadienal, β-ionone, 2-hexenal, ethyl salicylate, guaiacol, hexanal, hexanoic acid, linalool, methyl salicylate, nerol, phenylethyl alcohol, and tridecanal, classified as one acid, one alcohol, six aldehydes, one aromatic compound, two esters, and three terpenes (Supplementary Table S2, Figure S1). In addition, we detected significant differences among the plants with respect to the dominant volatile components, with methyl salicylate being identified as the most abundant compound in tomato flowers, whereas in both pepper and eggplant flowers, hexanal was found to be the primary volatile component (Figure 2, Table 1). To identify the fundamental chemical cues commonly released by the three nectar-producing crops, we implemented a filtering protocol. Although we initially identified 82, 63, and 60 VOCs in the flowers of tomato, pepper, and eggplant, respectively, in our EAG and behavioral analyses, we focused specifically on the 14 compounds present in all three species, with a view toward determining whether *A. mellifera* and *B. terrestris* have a generalized olfactory template encompassing diverse floral resources.

### 3.3. Electrophysiological Analysis of the Antennae of A. mellifera and B. terrestris

To verify whether the antennae of *A. mellifera* and *B. terrestris* can detect the 14 common compounds identified using GC-MS, we conducted EAG analysis. Test compounds prepared at four concentrations (0.1, 1, 10, and 100 µg/µL), along with solvent controls (hexane or ethanol), were used to stimulate the antennae of both species. The results indicated that in both *A. mellifera* and *B. terrestris*, the intensity of the response to each of the 14 compounds was higher than that to the solvent control (Supplementary Table S2). Moreover, for both species, we detected a dose–response relationship for the 14 compounds at 0.1 µg/µL, with increasing response intensity at higher concentrations. In *A. mellifera*, the response with the highest intensity was that to 100 µg/µL hexanoic acid [Mean (M), 533.08; standard error (SE), 13.68], whereas for bumblebees, the highest intensity response was that to 100 µg/µL methyl salicylate (M, 434.2; SE, 13.34).

**Figure 3:**
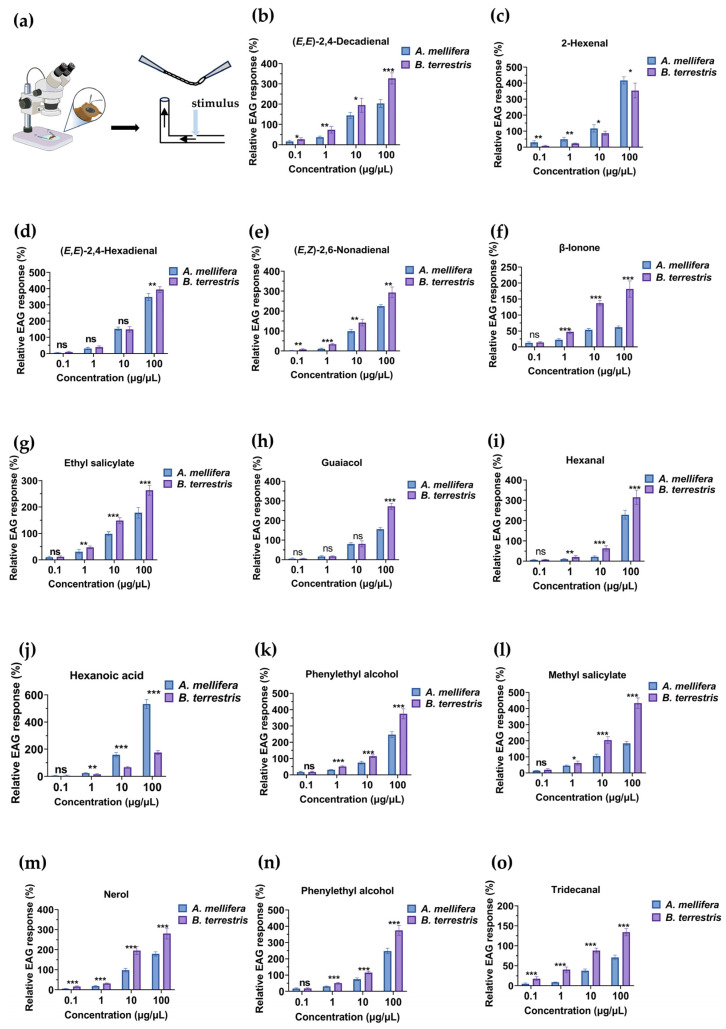
Electroantennographic (EAG) responses of *Apis mellifera* and *Bombus terrestris* to 14 common volatile organic compounds. (**a**) Schematic diagram of the EAG recording setup for bee antennae. (**b**–**o**) Dose–response EAG curves for the responses of *A. mellifera* and *B. terrestris* to the following synthetic compounds: (**b**) (*E*,*E*)-2,4-decadienal, (**c**) 2-hexenal, (**d**) (*E*,*E*)-2,4-hexadienal, (**e**) (*E*,*Z*)-2,6-nonadienal, (**f**) β-ionone, (**g**) ethyl salicylate, (**h**) guaiacol, (**i**) hexanal, (**j**) hexanoic acid, (**k**) linalool, (**l**) methyl salicylate, (**m**) nerol, (**n**) phenylethyl alcohol, and (**o**) tridecanal. Data are presented as the mean ± standard error. Asterisks indicate significant differences between species at a specific concentration (ns: *p* > 0.05; *: 0.01 < *p* < 0.05; **: 0.001 < *p* < 0.01; ***: *p* < 0.001).

Comparisons of responses to the same compounds at identical concentrations revealed that both two species showed significantly differences to ethyl salicylate (*p* = 0.002, *q* = 0.001, Cohen’s *d* = 1.65, 1 µg/µL; *p* < 0.001, *q* < 0.001, Cohen’s *d* = 3.72, 10 µg/µL; *p* < 0.001, *q* < 0.001, Cohen’s *d* = 3.11, 100 µg/µL), methyl salicylate (*p* = 0.023, *q* = 0.015, Cohen’s *d* = 1.24, 1 µg/µL; *p* < 0.001, *q* < 0.001, Cohen’s *d* = 4.05, 10 µg/µL; *p* < 0.001, *q* < 0.001, Cohen’s *d* = 7.14, 100 µg/µL), phenylethyl alcohol (*p* < 0.001, *q* < 0.001, Cohen’s *d* = 4.59, 1 µg/µL; *p* < 0.001, *q* < 0.001, Cohen’s *d* = 3.34, 10 µg/µL; *p* < 0.001, *q* < 0.001, Cohen’s *d* = 3.61, 100 µg/µL), hexanal (*p* = 0.007, *q* = 0.005, Cohen’s *d* = 1.39, 1 µg/µL; *p* < 0.001, *q* < 0.001, Cohen’s *d* = 3.07, 10 µg/µL; *p* < 0.001, *q* < 0.001, Cohen’s *d* = 2.11, 100 µg/µL), hexanoic acid (*p* = 0.008, *q* = 0.003, Cohen’s *d* = −1.35,1 µg/µL; *p* < 0.001, *q* < 0.001, Cohen’s *d* = −5.74, 10 μg/μL; *p* < 0.001, *q* < 0.001, Cohen’s *d* = −9.88, 100 μg/μL), and β-ionone (*p* < 0.001, *q* < 0.001, Cohen’s *d* = 3.47, 1 μg/μL; *p* < 0.001, *q* < 0.001, Cohen’s *d* = 8.65, 10 μg/μL; *p* < 0.001, *q* < 0.001, Cohen’s *d* = 4.66, 100 μg/μL) at concentrations of 1 μg/μL and above. *B. terrestris* showed higher electrophysiological sensitivity to ethyl salicylate, methyl salicylate, phenylethyl alcohol, hexanal, and β-ionone, whereas *A. mellifera* individuals were more sensitive to hexanoic acid. The two species also displayed differences in their sensitivity to (*E*,*E*)-2,4-hexadienal (*p* = 0.003, *q* = 0.009, Cohen’s *d* = 1.62) and guaiacol (*p* < 0.001, *q* < 0.001, Cohen’s *d* = 6.63) at 100 µg/µL, with *B. terrestris* showing greater sensitivity. In addition, we observed significant differences for linalool (*p* = 0.004, *q* = 0.007, Cohen’s *d* = 1.51, 0.1 μg/μL; *p* < 0.001, *q* = 0.001, Cohen’s *d* = 2.41, 1 μg/μL; *p* < 0.001, *q* < 0.001, Cohen’s *d* = 4.03, 10 μg/μL; *p* < 0.001, *q* < 0.001, Cohen’s *d* = 8.58, 100 μg/μL), nerol (*p* < 0.001, *q* < 0.001, Cohen’s *d* = 3.68, 0.1 μg/μL; *p* < 0.001, *q* < 0.001, Cohen’s *d* = 3.45, 1 μg/μL; *p* < 0.001, *q* < 0.001, Cohen’s *d* = 4.52, 10 μg/μL; *p* < 0.001, *q* < 0.001, Cohen’s *d* = 3.56, 100 μg/μL), (*E*,*E*)-2,4-decadienal (*p* = 0.02, *q* = 0.017, Cohen’s *d* = 1.19, 0.1 μg/μL; *p* < 0.001, *q* = 0.004, Cohen’s *d* = 2.12, 1 μg/μL; *p* = 0.01, *q* = 0.017, Cohen’s *d* = 1.33, 10 μg/μL; *p* < 0.001, *q* < 0.001, Cohen’s *d* = 3.66, 100 μg/μL), tridecanal (*p* < 0.001, *q* = 0.001, Cohen’s *d* = 2.38, 0.1 μg/μL; *p* < 0.001, *q* < 0.001, Cohen’s *d* = 4.78, 1 μg/μL; *p* < 0.001, *q* < 0.001, Cohen’s *d* = 6.65, 10 μg/μL; *p* < 0.001, *q* < 0.001, Cohen’s *d* = 5.82, 100 μg/μL), and (*E*,*Z*)-2,6-nonadienal (*p* < 0.001, *q* = 0.006, Cohen’s *d* = 1.57, 0.1 μg/μL; *p* < 0.001, *q* < 0.001, Cohen’s *d* = 4, 1 μg/μL; *p* < 0.001, *q* = 0.001, Cohen’s *d* = 2.23, 10 μg/μL; *p* < 0.001, *q* = 0.001, Cohen’s *d* = 2.48, 100 μg/μL) at concentrations of 0.1 µg/µL and above, with *B. terrestris* showing higher sensitivity than *A. mellifera*, the exception being 2-hexenal (*p* < 0.001, *q* = 0.007, Cohen’s *d* = −2.01, 0.1 μg/μL; *p* = 0.003, *q* = 0.007, Cohen’s *d* = −2.05, 1 μg/μL; *p* = 0.02, *q* = 0.024, Cohen’s *d* = −1.14, 10 μg/μL; *p* = 0.02, *q* = 0.024, Cohen’s *d* = −1.22, 100 μg/μL), to which *A. mellifera* was more sensitive. These findings accordingly provide evidence to indicate that differences in the antennal responses to linalool, nerol, (*E*,*E*)-2,4-decadienal, tridecanal, (*E*,*Z*)-2,6-nonadienal, and 2-hexenal may contribute to, or be associated with, differences observed in the behavior of *A. mellifera* and *B. terrestris* in response to solanaceous crops. Specifically, although the higher antennal sensitivity of *B. terrestris* to these compounds is taken to be indicative of a more efficient detection capacity, the behavioral valence of odorants is ultimately determined by central neural processing, and antennal sensitivity alone does not directly dictate attraction or repellence (Supplementary Table S3). Consequently, the functional role of these specific volatiles in driving the distinct visitation patterns remains to be validated based on further behavioral assays (Table 2, Figure 3).

### 3.4. Behavioral Preferences of A. mellifera and B. terrestris for Common Emitted by Three Solanaceous Crop Plants

**Figure 4:**
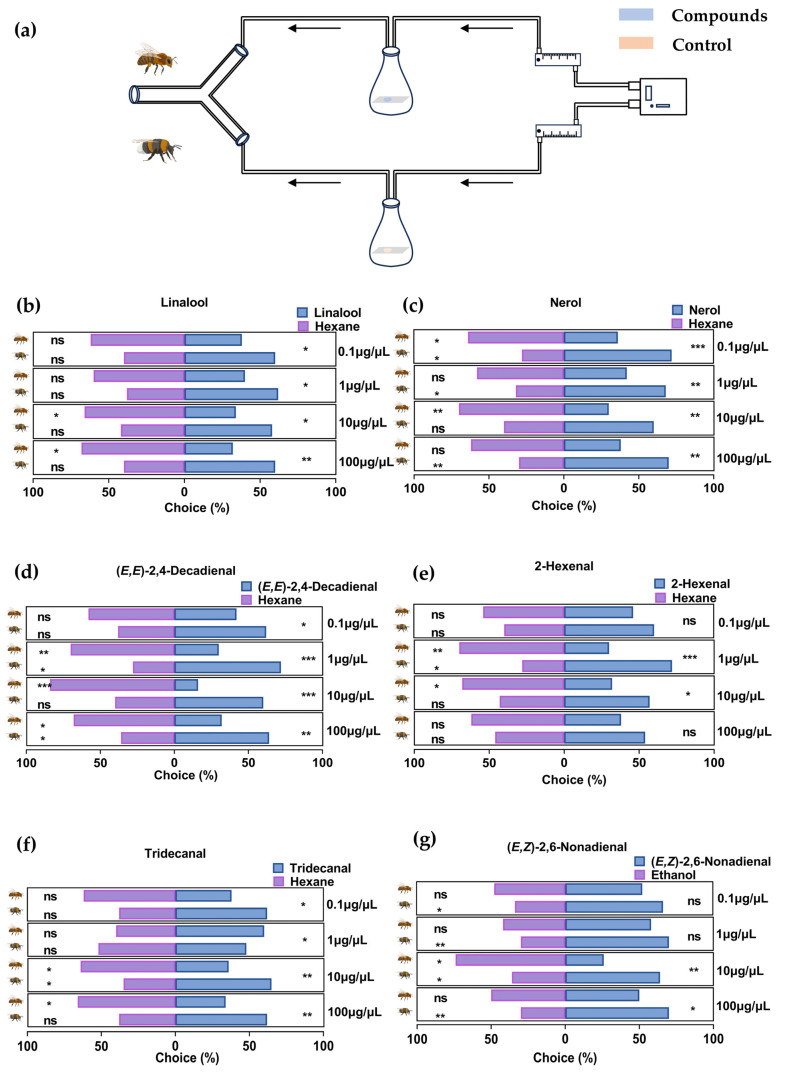
Behavioral responses of *Apis mellifera* and *Bombus terrestris* to six synthetic volatile compounds. (**a**) Schematic representation of a Y-tube olfactometer illustrating the direction of airflow, the treatment arm containing the stimulus, and the control arm containing the solvent. (**b**–**g**) Behavioral preferences of *Apis mellifera* and *Bombus terrestris* toward (**b**) linalool, (**c**) nerol, (**d**) (*E*,*E*)-2,4-decadienal, (**e**) 2-hexenal, (**f**) tridecanal, and (**g**) (*E*,*Z*)-2,6-nonadienal. (ns: *p* > 0.05; *: 0.01 < *p* < 0.05; **: 0.001 < *p* < 0.01; ***: *p* < 0.001).

When presented at a concentration of 0.1 μg/μL, we detected no significant differences regarding the responses of *A. mellifera* and *B. terrestris* to 2-hexenal or (*E*,*Z*)-2,6-nonadienal. However, significant behavioral divergence between the two species was observed with respect to linalool (*p* = 0.03), nerol (*p* < 0.001), (*E*,*E*)-2,4-decadienal (*p* = 0.04), and tridecanal (*p* = 0.02). At a concentration of 1 μg/μL, whereas differences in the response toward (*E*,*Z*)-2,6-nonadienal remain non-significant, that toward the other five assessed compounds reached the level of significance (*p* < 0.05), notably with respect to (*E*,*E*)-2,4-decadienal and 2-hexenal, for which we detected highly significant differences (*p* < 0.001). At a higher concentration of 10 μg/μL, *A. mellifera* and *B. terrestris* showed significant behavioral differences toward all six of the assessed compounds (*p* < 0.05), whereas, when the six compound were presented at a concentration of 100 μg/μL, significant differences remained for five compounds (*p* < 0.05), with the exception being the response toward (*E*,*Z*)-2,6-nonadienal. Behavioral assays revealed contrasting responses between *A. mellifera* and *B. terrestris* toward several of the assessed VOCs. Specifically, for *A. mellifera*, we detected significant reduction in attraction to one or more concentrations of linalool (1 and 100 μg/μL), nerol (0.1 and 10 μg/μL), (*E*,*E*)-2,4-decadienal (1, 10, and 100 μg/μL), 2-hexenal (1 and 10 μg/μL), tridecanal (10 and 100 μg/μL), and (*E*,*Z*)-2,6-nonadienal (10 μg/μL). Conversely, *B. terrestris* showed significant attraction to nerol (0.1, 1, and 100 μg/μL), (*E*,*E*)-2,4-decadienal (1 and 100 μg/μL), 2-hexenal (1 μg/μL), tridecanal (10 μg/μL), and (*E*,*Z*)-2,6-nonadienal (0.1, 1, 10, and 100 μg/μL). Notably, when presented at the same concentrations, certain compounds elicited contrasting behavioral tendencies, (significant avoidance in *A. mellifera* and attraction in *B. terrestris*), including nerol at 0.1 μg/μL (*p* = 0.048 for *A. mellifera*; *p* = 0.02 for *B. terrestris*), *(E*,*E*)-2,4-decadienal at 1 μg/μL (*p* = 0.005 for *A. mellifera*; *p* = 0.02 for *B. terrestris*) and 100 μg/μL (*p* = 0.011 for *A. mellifera*; *p* = 0.048 for *B. terrestris*), 2-hexenal at 1 μg/μL (*p* = 0.005 for *A. mellifera*; *p* = 0.02 for *B. terrestris*), tridecanal at 10 μg/μL (*p* = 0.048 for *A. mellifera*; *p* = 0.048 for *B. terrestris*), and (*E*,*Z*)-2,6-nonadienal at 10 μg/μL (*p* = 0.048 for *A. mellifera*; *p* = 0.048 for *B. terrestris*). These findings thus indicate that these VOCs could serve as the primary chemical cues underlying the observed differences in the foraging behaviors of *A. mellifera* and *B. terrestris* when visiting the flowers of solanaceous plants (Figure 4).

## 4. Discussion

Floral volatiles, which constitute the chemical communication system of flowers, play essential roles in attracting pollinators and facilitating pollination. Although numerous previous studies have identified VOCs in the flowers of crop plants that influence pollinator behavior, research on differences in the pollination efficiencies of different pollinators of key crops, such as those in the family Solanaceae, remains limited. In this study, we investigated mechanisms underlying differences in the pollination behavior of *A. mellifera* and *B. terrestris* toward the flowers of three solanaceous crop plants from a chemoecological perspective, and sought to identify the VOCs contributing to differences in the floral visitation behaviors of these two pollinators.

Our findings revealed clear differential preferences between *A. mellifera* and *B. terrestris* for flowers of the three solanaceous crop plants. *A. mellifera* showed a significantly lower preference for floral volatiles emitted by tomato plants, thereby providing evidence of a weak olfactory attraction. These findings are consistent with those obtained by Liu et al., who likewise reported a bumblebee preference for tomato flowers [46]. To ensure standardization, we used active foragers, which, due to neuromodulatory changes and sensory maturation associated with foraging in Hymenoptera, exhibit more stable olfactory responses than younger nest bees. Thus, selecting only active foraging females minimized interference from age-related shifts and previous experience in our EAG and behavioral assays. Furthermore, the use of large samples of bees from multiple queenright colonies ensured that the observed differential responses of *A. mellifera* and *B. terrestris* are representative of species-specific chemical ecology rather than individual histories. However, Solanaceae species require buzz pollination for effective pollen release. *B. terrestris* can perform it, but *A. mellifera* cannot. Floral volatiles may act as an olfactory filter complementing these mechanical constraints; honeybee avoidance prevents visits to flowers with inaccessible pollen, optimizing foraging. Consequently, divergent VOC responses are part of a multimodal partitioning strategy between these two bee species.

Using GC-MS analyses, we identified 14 VOCs commonly produced by all three solanaceous crops. Although our use of liquid nitrogen and SPME may have included endogenous metabolites, future research using dynamic headspace sampling on live intact flowers is needed to more precisely represent real-time floral scent emission. Notably, whereas olefins were identified as the most abundant volatiles in tomato flowers, aldehydes were found to predominate in the flowers of pepper and eggplant. The identification of hexanal is consistent with the findings of Meijer et al. [47]. Previous studies have confirmed that tomato flowers and fruits emit over 400 types, including aldehydes, alcohols, ketones, esters, terpenes, and sulfur-containing compounds [46,47,48]. Core types of tomato floral VOCs include terpenoids, aldehydes, and alcohols, among which compounds such as β-phellandrene, (+)-2-carene, α-pinene, *p*-cymene, and β-caryophyllene have been established to play key roles in chemical communication and potentially serve as cues for pollinator olfactory recognition. The core VOCs detected in tomato flowers, such as (+)-4-carene, α-phellandrene, *p*-cymene, and caryophyllene, are consistent with those described in the previous literature, thus enhancing the credibility of our findings in this study. Eggplant volatiles have been established to predominantly comprise terpenes and aldehydes, with α-humulene, (*E*)-β-caryophyllene, and α-pinene shown to attract whiteflies [49], whereas compounds such as *p*-xylene, decane, and benzene in peppers have been identified as attractants of the Chinese aphid parasitoid wasp *Aphelinus varipes* [50]. However, the types of volatiles detected in these crop plants can differ, owing to differences in plant cultivars, growth environments, and the time of harvest. In our analyses, we focused on the 14 commonly detected VOCs to identify the core olfactory template, as these represent the most stable and prominent chemical signals. However, each species also emitted many unique VOCs (e.g., 62 in tomato). Consequently, we plan to systematically investigate crop-specific unique VOCs to better understand floral constancy and pollinator discrimination.

We tested floral volatile concentrations from 0.1 to 100 µg/µL to characterize honeybee dose–response dynamics. Although these concentrations may not match natural emission rates, they cover a broad range given airborne dilution and variable forager sensitivities. Future studies should quantify actual headspace concentrations for field-realistic interpretation. EAG assays showed that both *A. mellifera* and *B. terrestris* show concentration-dependent physiological responses to all 14 of the assessed synthetic compounds, confirming their broad olfactory spectrum for detecting host-plant volatiles. These compounds, including aldehydes [hexanal and (*E*,*E*)-2,4-decadienal], terpenoids (nerol and linalool), and esters (methyl salicylate), are commonly found in Solanaceae and other entomophilous plants. Our findings are consistent with and extend previous research on the olfactory perception of hymenopteran pollinators. For example, linalool, a common monoterpene alcohol and potent ligand for odorant receptors in *A. mellifera*, has been established to influence floral constancy and the foraging efficiency of these honeybees [51]. Our findings confirmed the complexity of the relationship between volatiles and pollinators, with the acute sensitivity toward 2-hexenal detected in this study being consistent with the findings reported by Zhang et al. [42], who identified green leaf volatiles (GLVs) as essential signals for *B. terrestris* during long-distance floral orientation. The 14 tested compounds universally elicited electrophysiological responses in both bee species, underscoring the complexity of plant–pollinator chemical communication. Although their sensory ranges overlapped, *A. mellifera* and *B. terrestris* differed in response thresholds and peak EAG amplitudes. Notably, six compounds—linalool, nerol, (*E*,*E*)-2,4-decadienal, 2-hexenal, tridecanal, and (*E*,*Z*)-2,6-nonadienal—consistently evoked significantly different EAG responses between the two species at all concentrations tested. However, EAG responses observed represent peripheral olfactory detection. Although higher EAG amplitudes do not guarantee behavioral valence, which is processed centrally. Furthermore, our present findings lack molecular evidence. Therefore, the link between electrophysiological sensitivity and behavioral outcomes remains merely correlative. Accordingly, further research involving RNA interference or CRISPR/Cas9-mediated gene editing will be necessary to establish a direct causal link.

The findings of our behavioral assays revealed a clear divergence in the response patterns of *A. mellifera* and *B. terrestris* toward specific VOCs, the most striking of which were the reciprocal activities observed at identical concentrations of several compounds, including nerol, (*E*,*E*)-2,4-decadienal, 2-hexenal, tridecanal, and (*E*,*Z*)-2,6-nonadienal. Whereas these volatiles evoked a significant avoidance response in *A. mellifera*, they were found to serve as potent attractants for *B. terrestris*, thereby providing evidence that plant volatiles can act as chemical filters that differentially regulate the recruitment of these two major pollinator species. From a chemoecological perspective, this behavioral specificity may stem from evolutionary differences in perceiving defensive and nutritional signals. For example, GLVs such as 2-hexenal are typically associated with plant tissue damage or herbivory [52]. We speculate that 2-hexenal, constitutively emitted by solanaceous plants even under non-stressed conditions, serves as a high-contrast chemical marker for generalist bumblebees, enhancing plant detectability against complex olfactory backgrounds and signaling healthy resources for efficient foraging. Thus, the avoidance behavior of *A. mellifera* may represent a survival strategy to evade induced plant defenses or low-quality floral resources, whereas the attraction of *B. terrestris* could be linked to the higher environmental plasticity and specialized foraging strategies for specific host plants, such as those in the family Solanaceae. Bumblebees may, thus, utilize these signals for long-distance orientation [42]. Furthermore, the concentration-dependent effects observed in this study may represent a further layer of complexity modulating chemical communication. Although we detected notable differences in the behavioral responses elicited by all six compounds assessed at 10 μg/μL, the disparities in responses to certain compounds [2-hexenal and (*E*,*Z*)-2,6-nonadienal] were observed to be less pronounced at lower or extremely high concentrations. Interestingly, although the findings of previous studies have indicated that linalool acts as an attractant for *A. mellifera* [53,54], we found that these bees show a less conspicuous preference at higher concentrations (10 μg/μL and 100 μg/μL), which thus tends to indicate that rather than being hard-wired responses, pollinator olfactory preferences are instead stringently regulated by scent concentration gradients. In addition, the olfactory responses in bees are modulated by both genetic factors and previous experience. Differences from previous studies may reflect variations, and field-foraged bees likely learned their responses to linalool. The diminished preference observed at high concentrations might thus reflect a learned avoidance or a sensory saturation effect, thereby reinforcing the idea that pollinator attraction is a plastic trait shaped by both innate biology and environmental interactions. Ruiz-Hernández et al. showed that behavioral preferences are concentration-dependent [55]. For *o*-acetanisole, bumblebees showed significant avoidance at a high concentration of 1000 ppm, whereas no significant responses were detected at lower concentrations (10 or 100 ppm). Similarly, He et al. demonstrated that bumblebees show positive chemotaxis toward α-terpineol at concentrations of 0.1 and 1 μg/μL, whereas this compound elicits avoidance at 100 μg/μL [56].

Our findings are based on specific varieties of solanaceous crop plants; other cultivars may differ in volatile profiles, altering honeybee attraction. Our bioassays identified several key volatiles that individually elicit significant behavioral responses, but in nature, these compounds occur in complex mixtures where synergistic or antagonistic interactions matter. Accordingly, the single-compound approach is only a preliminary step in characterizing pollinator preferences; future studies should use EAG-GC-MS with synthetic mixtures and validate results in natural environments where visual cues and other factors matter. Moreover, laboratory findings require validation in natural environments. Greenhouse-scale volatile augmentation studies are needed to confirm ecological relevance.

## 5. Conclusions

In summary, our findings in this study revealed the strikingly contrasting behavioral responses of two species of hymenopteran pollinators to three species of crop plants in the family Solanaceae, with *B*. *terrestris* being characterized by pronounced floral attraction, whereas *A*. *mellifera* showed no clear host-plant preferences. Of the 14 floral volatiles commonly emitted by the three crop plants, six elicited distinct electrophysiological responses in bees, five of which appear to play a central role in mediating the observed interspecific differences in floral attraction. Collectively, our findings facilitated the identification of the core chemical cues that determine pollinator foraging preferences for solanaceous crops and will serve as a chemoecological basis for determining the disparities in pollination efficiencies of the two bee species for these crops. From an agricultural perspective, these findings will provide a strategic framework for precision pollination management. Our identification of the chemical cues that contribute to attracting *B. terrestris* could provide opportunities for exploiting the volatile profiles of solanaceous crops, either through selective breeding or the development of targeted synthetic lures, to optimize pollinator recruitment. Such strategies could be adopted to substantially enhance fruit set and yield in both greenhouse and open-field environments, particularly in the case of crops for which the pollination efficiency of *A. mellifera* is limited.

## Figures and Tables

**Table 1:** Chemical composition and relative abundance of the common volatile organic compounds produced by three solanaceous crop plants.

Class	Compounds	CAS	Relative Content (%)		
			*S. lycopersicum*	*C. annuum*	*S. melongena*
Aldehydes	(*E*,*E*)-2,4-decadienal	25152-84-5	0.033 ± 0.007	0.04 ± 0.044	0.633 ± 0.14
	*(E*,*E*)-2,4-hexadienal	142-83-6	0.347 ± 0.074	0.27 ± 0.098	0.437 ± 0.146
	(*E*,*Z*)-2,6-nonadienal	557-48-2	0.055 ± 0.015	0.27 ± 0.052	0.4 ± 0.157
	2-hexenal	505-57-7	11.883 ± 6.42	10.555 ± 2.114	17.61 ± 5.926
	Hexanal	66-25-1	5.807 ± 2.207	36.015 ± 28.914	20.077 ± 11.096
	Tridecanal	10486-19-8	0.01 ± 0	0.01 ± 0	0.01 ± 0
Terpenoids	Linalool	78-70-6	0.133 ± 0.015	0.027 ± 0.006	0.16 ± 0
	Nerol	106-25-2	0.137 ± 0.019	0.037 ± 0.015	0.023 ± 0.006
	β-ionone	79-77-6	0.065 ± 0.005	0.037 ± 0.015	0.043 ± 0.006
Esters	methyl salicylate	119-36-8	16.273 ± 0.807	10.273 ± 1.133	7.233 ± 1.964
	ethyl salicylate	118-61-6	0.353 ± 0.122	2.43 ± 0.245	2.397 ± 0.725
Acids	hexanoic acid	142-62-1	0.05 ± 0	0.29 ± 0.085	0.42 ± 0
Alcohols	phenylethyl alcohol	60-12-8	0.043 ± 0.012	0.027 ± 0.021	0.067 ± 0.006
Aromatics	Guaiacol	90-05-1	0.617 ± 0.219	0.263 ± 0.098	0.06 ± 0.017

Mean species-level abundance ± standard error for each compound.

**Table 2:** Fourteen synthetic standards used for electroantennography and Y-tube bioassays.

No.	Compounds	Purity (%)	Origin
1	(*E*,*E*)-2,4-decadienal	≥90.0	TCI ^1^
2	(*E*,*E*)-2,4-hexadienal	≥95.0	Aladdin ^2^
3	(*E*,*Z*)-2,6-nonadienal	≥95.0	TCI
4	2-hexenal	≥97.0	TCI
5	hexanal	≥98.0	TCI
6	tridecanal	≥95.0	TCI
7	linalool	≥96.0	TCI
8	nerol	≥98.0	TCI
9	β-ionone	≥95.0	TCI
10	methyl salicylate	≥99.0	TCI
11	ethyl salicylate	≥99.0	TCI
12	hexanoic acid	≥98.0	TCI
13	phenylethyl alcohol	≥98.0	TCI
14	guaiacol	≥98.0	TCI

^1^ TCI: Tokyo, Japan; ^2^ Aladdin: Shanghai, China.

## Data Availability

The original contributions presented in the study are included in the article/Supplementary Materials. Further inquiries can be directed to the corresponding authors.

## Supplementary Materials

The following supporting information can be downloaded at: https://www.mdpi.com/article/10.3390/insects17050507/s1, Table S1: Chemical composition of VOCs from three Solanaceous crops; Table S2: EAG Responses of *A. mellifera* and *B. terrestris* to 14 synthetic compounds; Table S3: Relationship between the intensity of the electroantennographic (EAG) responses and behavioral preferences of *Apis mellifera* and *Bombus terrestris* for six selected volatile compounds presented at different concentrations; Figure S1: Total Ion Chromatogram (TIC) of Tomato, Pepper, and Eggplant Samples.
