# Supplementary material for: Olfactory Responses of Apis mellifera and Bombus terrestris to Floral Volatiles from Three Solanaceae Crops
Source: Insects. 2026 May 15;17(5):507. doi: 10.3390/insects17050507 (PMC13207054; doi:10.3390/insects17050507)
Supplement: Supplementary file 1 [file insects-17-00507-s001.zip › insects-4182464-supplementary.pdf]

Supplementary Materials

Table S1. Chemical composition of VOCs from three Solanaceous crops

Compounds	CAS	*S. lycopersicum*		*C. annuum*		*S. melongena*	
		Retain Time (min)	Relative Content (%)	Retain Time (min)	Relative Content (%)	Retain Time (min)	Relative Content (%)
(+)-4-Carene	29050-33-7	15.398	0.385	-	-	-	-
(1*S*,2*R*,4*R*,7*R*)-4-Isopropyl-7-methyl-3,8-dioxatricyclo[5.1.0.02,4]octane	1619-26-7	21.119	0.043	-	-	-	-
(3*E*,7*E*)-4,8,12-Trimethyltrideca-1,3,7,11-tetraene	62235-06-7	-	-	25.591	0.033	-	-
(3*R*,4a*S*,5*R*)-4a,5-Dimethyl-3-(prop-1-en-2-yl)-1,2,3,4,4a,5,6,7-octahydronaphthalene	24741-64-8	23.878	0.045	-	-	-	-
(*E*)-2-Heptenal	18829-55-5	-	-	-	-	10.597	0.593
(*E*)-2-Hexenal	6728-26-3	5.137	5.145	-	-	4.686	14.330
(*E*)-2-Nonenal	18829-56-6	-	-	-	-	17.487	0.777
(*E*)-2-Octen-1-ol	18409-17-1	-	-	-	-	14.723	0.020
(*E*)-2-Octenal,	2548-87-0	-	-	14.425	0.047	14.439	3.233
(*E*)-4,8-Dimethylnona-1,3,7-triene	19945-61-0	-	-	16.161	0.080	-	-
(*E*)-5-Decen-1-ol	56578-18-8	-	-	20.034	0.030	-	-
(*E*)-Cinnamaldehyde	14371-10-9	-	-	20.445	0.130	-	-
(*E*,*E*)-1,5-Cyclodecadiene, 1,5-dimethyl-8-(1-methylethylidene)	15423-57-1	23.520	0.270	-	-	-	-
(*E*,*E*)-2,4-Decadienal	25152-84-5	20.925	0.033	20.918	0.040	20.918	0.633
(*E*,*E*)-2,4-Heptadienal	4313-03-5	-	-	12.578	0.070	12.218	0.583
(*E*,*E*)-2,4-Hexadienal	142-83-6	8.237	0.347	8.298	0.270	8.282	0.437
(E,E)-2,4-Nonadienal	5910-87-2	-	-	-	-	18.983	0.477
(*E*,*E*)-2,6-Dimethyl-1,3,5,7-octatetraene	460-01-5	-	-	16.622	0.050	-	-
(*E*,*Z*)-2,4-Decadienal	25152-83-4	21.432	0.010	-	-	-	-
(E,Z)-2,6-Nonadienal	557-48-2	17.309	0.055	17.289	0.270	17.278	0.400
(*R*)-4-Hexen-1-ol, 5-methyl-2-(1-methylethenyl)	498-16-8	-	-	17.599	0.045	17.587	0.055
(S)-1-Hexanol, 4-methyl	1767-46-0	-	-	-	-	10.129	1.127
(*S*)-7-Octen-1-ol, 3,7-dimethyl-	6812-78-8	19.262	0.097	-	-	-	-
(*Z*)-1,3,6-Octatriene, 3,7-dimethyl	3338-55-4	14.041	0.475	-	-	-	-
(*Z*)-2-Cyclopenten-1-one, 3-methyl-2-(2-pentenyl)	488-10-8	-	-	22.853	0.283	-	-
(*Z*)-2-Decenal	2497-25-8	-	-	-	-	20.163	0.060
(*Z*)-2-Decene, 9-methyl	74630-24-3	-	-	19.507	0.015	-	-
(*Z*)-2-Penten-1-ol	1576-95-0	-	-	-	-	0.036	0.465
(*Z*)-3,6-Octadien-1-ol, 3,7-dimethyl	5944-20-7	-	-	19.597	0.027	-	-
(*Z*)-5,9-Undecadien-2-one, 6,10-dimethyl	3879-26-3	-	-	-	-	23.731	0.070
(*Z*)-Hex-3-enyl (*E*)-2-methylbut-2-enoate	67883-79-8	-	-	21.488	0.027	-	-

1,12-Tridecadiene	21964-48-7	17.937	2.815	-	-	-	-
1,1'-Bicyclopropyl, 2,2,2',2'-tetramethyl	68998-20-9	-	-	16.796	0.017	16.730	0.040
1,2-Cyclohexanediol, 1-methyl-4-(1-methylethenyl)	1946-00-5	21.979	0.037	-	-	-	-
1,3,5-Cycloheptatriene, 3,7,7-trimethyl	3479-89-8	11.095	1.227	-	-	-	-
1,3-Cyclohexadiene-1-methanol, 4-(1-methylethyl)	1413-55-4	20.663	0.035	-	-	-	-
1,3-Cyclopentadiene, 1,2,3,4-tetramethyl-5-methylene	76089-59-3	14.279	0.105	-	-	-	-
1,4-Heptadiene, 3-methyl	1603-01-6	-	-	-	-	14.332	0.095
1,4-Hexadiene, 3-ethyl	2080-89-9	-	-	12.501	0.165	-	-
1,5-Cyclohexadiene-1-methanol, 4-(1-methylethyl)	19876-45-0	20.442	0.025	-	-	-	-
14-Hydroxycaryophyllene	50277-33-3	26.780	0.043	-	-	-	-
1-Cyclohexene-1-carboxaldehyde, 2,6,6-trimethyl	432-25-7	-	-	19.103	0.043	19.085	0.070
1-Cyclohexene-1-carboxaldehyde, 4-(1-methylethyl)	21391-98-0	20.588	0.047	-	-	-	-
1H,3H-Furo[3,4-c]furan, tetrahydro	5175-36-0	-	-	-	-	17.116	0.040
1-Hexanol	111-27-3	5.782	0.055	6.003	1.100	-	-
1-Hexanol, 4-methyl	818-49-5	10.105	0.097	-	-	-	-
1-Methyl-1H-pyrazolo[3,4-b]pyridin-3-ylamine	72583-83-6	23.081	0.063	23.071	0.037	-	-
1-Octen-3-ol	3391-86-4	11.609	0.230	-	-	11.630	0.350
1-Octen-3-one	4312-99-6	-	-	-	-	11.481	0.057
1-Pentanol, 4-methyl-	626-89-1	3.415	0.283	-	-	-	-
2,2-Dimethyl-3-heptanone	19078-97-8	-	-	14.526	0.070	-	-
2,3-Octanedione	585-25-1	-	-	-	-	11.868	0.160
2,4-Decadienal	2363-88-4	-	-	21.423	0.035	21.425	0.315
2-Caren-10-al	6909-19-9	20.783	0.050	-	-	-	-
2-Cyclohexen-1-ol, 1-methyl-4-(1-methylethenyl)-, trans	7212-40-0	16.393	0.007	-	-	-	-
2-Cyclohexen-1-ol, 3-methyl-6-(1-methylethyl)-, cis	16721-38-3	18.846	0.015	-	-	-	-
2-Cyclohexen-1-one, 3-methyl-6-(1-methylethyl)	89-81-6	20.001	0.243	-	-	-	-
2-Hexenal	505-57-7	4.573	11.883	4.826	10.555	4.791	17.610
2-Hexene, 3,5,5-trimethyl	26456-76-8	-	-	-	-	11.316	0.035
2-Hexenoic acid	1191-04-4	-	-	13.162	0.020	-	-
2-Octenal, 2-butyl	13019-16-4	-	-	22.525	0.037	-	-
2-Pentadecanone, 6,10,14-trimethyl	502-69-2	28.797	0.020	-	-	-	-
2-Propen-1-ol, 3-phenyl	104-54-1	-	-	21.165	0.257	-	-
3,5-Octadien-2-one	38284-27-4	-	-	-	-	14.769	0.117
3,6-Dimethyl-2,3,3a,4,5,7a-hexahydrobenzofuran	70786-44-6	18.255	0.387	-	-	-	-
3-Buten-2-one, 4-(2,2,6-trimethyl-7-oxabicyclo[4.1.0]hept-1-yl)	23267-57-4	-	-	24.317	0.015	24.312	0.010
3-Octen-2-one	1669-44-9	-	-	-	-	13.722	0.010
4-Cyanocyclohexene	100-45-8	-	-	-	-	12.953	0.200

4-Isopropyl-1-methylcyclohex-2-enol	619-62-5	16.483	0.060	-	-	-	-
4-Oxohex-2-enal	20697-55-6	10.793	0.050	10.826	0.127	-	-
5,8-Dimethylenebicyclo[2.2.2]oct-2-ene	29845-13-4	15.427	0.080	-	-	-	-
5,9-Undecadien-2-ol, 6,10-dimethyl	53837-34-6	23.802	0.067	-	-	-	-
5,9-Undecadien-2-one, 6,10-dimethyl	689-67-8	23.745	0.780	-	-	-	-
5-Hepten-2-one, 6-methyl	110-93-0	-	-	-	-	11.794	0.243
6-Octen-1-ol, 7-methyl-3-methylene	13066-51-8	19.015	0.333	-	-	-	-
7-Oxabicyclo[4.1.0]heptane, 1-methyl-4-(1-methylethenyl)	1195-92-2	16.724	1.067	-	-	-	-
8-Methylnonanoic acid	5963-14-4	-	-	-	-	29.978	0.010
Aromandendrene	489-39-4	25.541	0.020	-	-	-	-
Benzaldehyde	100-52-7	10.615	0.085	10.654	0.040	-	-
Benzene, 1-methyl-3-(1-methylethyl)	535-77-3	13.553	0.365	-	-	-	-
Benzene, 4-ethyl-1,2-dimethyl	934-80-5	14.848	0.325	-	-	-	-
Benzeneacetaldehyde	122-78-1	-	-	13.876	0.025	13.878	0.370
Benzoic acid, 2-hydroxy-, phenylmethyl ester	118-58-1	-	-	29.228	0.010	-	-
Benzoic acid, ethyl ester	93-89-0	-	-	17.780	0.113	-	-
Benzoic acid, hexyl ester	6789-88-4	-	-	25.701	0.010	-	-
Benzyl alcohol	100-51-6	-	-	-	-	13.544	1.033
Bicyclo[2.2.1]heptan-2-one, 4,7,7-trimethyl-, (1*S*)	10292-98-5	17.763	0.883	-	-	-	-
Bicyclo[3.1.0]hex-2-ene, 4-methylene-1-(1-methylethyl)	36262-09-6	14.137	0.123	-	-	-	-
Bicyclo[3.1.0]hexane, 4-methylene-1-(1-methylethyl)	3387-41-5	13.532	19.095	-	-	-	-
Bicyclo[4.1.0]hept-2-ene, 3,7,7-trimethyl-, (1*S*-cis)	4497-92-1	12.267	4.587	-	-	-	-
b-Ionone	79-77-6	24.272	0.065	24.269	0.037	24.265	0.043
b-Myrcene	123-35-3	11.972	0.743	-	-	-	-
b-Ocimene	13877-91-3	-	-	14.933	1.330	-	-
Carane, 4,5-epoxy-, trans	6909-20-2	16.127	0.157	-	-	-	-
Caryophylla-4(12),8(13)-dien-5α-ol	19431-79-9	26.566	0.033	-	-	-	-
Caryophyllene	87-44-5	23.430	3.803	-	-	-	-
Caryophyllene oxide	1139-30-6	25.862	0.190	-	-	-	-
cis-3-Hexenyl salicylate	65405-77-8	-	-	26.872	0.010	-	-
cis-4,5-Epoxy-(E)-2-decenal	188430-50-6	-	-	22.593	0.050	22.480	0.040
Copaene	3856-25-5	22.613	0.073	-	-	-	-
Cyclohexane, 1-butenylidene	36144-40-8	-	-	20.613	0.010	20.404	0.157
Cyclohexane, 1-ethenyl-1-methyl-2,4-bis(1-methylethenyl)-, [1S-(1.alpha.,2.beta.,4.beta.)]	515-13-9	22.841	0.427	-	-	-	-
Cyclohexanone, 2-methyl-5-(1-methylethenyl)-, trans	1478602	18.605	0.303	-	-	-	-
Cyclohexene, 1-methyl-4-(1-methylethylidene)	586-62-9	16.240	0.100	-	-	-	-
Cyclohexene, 4-ethenyl-4-methyl-3-	20307-84-0	21.824	1.690	-	-	-	-

(1-methylethenyl)-1-(1-methylethyl)-, (3R-trans)							
Cyclopentane, 1-ethyl-1-methyl-	16747-50-5	-	-	-	-	14.030	0.540
Cyclopentaneacetaldehyde, 2-formyl-3-methyl-α-methylene	5951-57-5	-	-	23.215	0.020	-	-
Decyl octyl ether	63559-00-6	-	-	-	-	26.734	0.017
Dodecane	112-40-3	-	-	24.553	0.027	-	-
ethyl salicyclate	118-61-6	20.338	0.353	20.378	2.430	20.347	2.397
Formic acid, 3-methylbut-2-yl ester	1334-74-3	-	-	10.011	0.020	9.977	0.020
Germacrene D	23986-74-5	24.388	0.180	-	-	-	-
gulaiacol	90-05-1	15.228	0.617	15.221	0.263	15.215	0.060
Heneicosane, 11-(1-ethylpropyl)	55282-11-6	-	-	25.359	0.010	-	-
Heptanal	111-71-7	-	-	7.941	0.060	7.919	0.213
Heptane, 1,1'-oxybis	629-64-1	-	-	-	-	15.314	0.075
Hexadecane	544-76-3	-	-	22.961	0.040	22.954	0.035
Hexanal	66-25-1	1.268	5.807	1.532	36.015	1.409	20.077
Hexane, 2,4,4-trimethyl	16747-30-1	-	-	-	-	22.813	0.010
Hexane, 3,3,4-trimethyl	16747-31-2	-	-	-	-	26.662	0.010
Hexane, 3-methyl-4-methylene	3404-67-9	10.445	0.037	-	-	-	-
Hexanoic acid	142-62-1	11.832	0.050	12.396	0.290	12.363	0.420
Hexyl tiglate	16930-96-4	-	-	21.624	0.027	-	-
Humulene	6753-98-6	24.002	1.270	-	-	-	-
Humulene epoxide I	19888-33-6	26.078	0.057	-	-	-	-
Isospathulenol	88395-46-4	26.413	0.057	-	-	-	-
Linalool	78-70-6	15.767	0.133	15.769	1.600	15.750	0.160
Methyl anthranilate	134-20-3	-	-	21.883	0.007	-	-
Methyl salicylate	119-36-8	18.557	16.273	18.541	10.273	18.425	7.233
Myroxide	28977-57-3	-	-	16.901	0.155	-	-
Naphthalene, 1,2,3,5,6,8a-hexahydro-4,7-dimethyl-1-(1-methylethyl)-, (1S-cis)	483-76-1	24.899	0.020	-	-	-	-
Naphthalene, 2,3,4,4a,5,6-hexahydro-1,4a-dimethyl-7-(1-methylethyl)	473-14-3	24.451	0.023	-	-	-	-
Nerol	106-25-2	19.857	0.137	19.864	0.037	19.845	0.023
Nonadecane	629-92-5	-	-	26.737	0.010	-	-
Nonane, 2,2,4,4,6,8,8-heptamethyl	4390-04-9	-	-	22.656	0.010	22.653	0.035
Octadecane	593-45-3	-	-	27.187	0.030	-	-
Octanoic acid	124-07-2	-	-	-	-	17.714	0.065
Oxime-, methoxy-phenyl-	3358-18-7	-	-	-	-	8.136	0.220
Oxirane, hexyl-	2984-50-1	-	-	-	-	15.896	0.495
p-Cymen-7-ol	536-60-7	20.863	0.033	-	-	-	-
p-Cymene	99-87-6	13.222	1.065	-	-	-	-
Pentadecane, 2,6,10,14-tetramethyl	1921-70-6	-	-	27.224	0.020	-	-
Phenol, 2-methoxy-4-(2-propenyl)-, acetate	93-28-7	22.092	0.097	-	-	-	-
Phenylethyl Alcohol	60-12-8	16.071	0.043	16.071	0.027	16.056	0.067
p-Menth-2-en-7-ol, cis	19898-86-3	20.192	0.080	-	-	-	-
Pyrazine, 2-methoxy-3-(2-methylpropyl)	24683-00-9	-	-	17.917	0.093	-	-
Tetradecane, 2,5-dimethyl	56292-69-4	22.664	0.015	-	-	-	-

trans-2-Dodecen-1-ol	69064-37-5	-	-	-	-	13.449	1.730
trans-Carveol	1197-07-5	19.101	0.203	-	-	-	-
trans-α-Bergamotene	13474-59-4	-	-	-	-	23.564	0.015
trans-α-Ocimene	3779-61-1	-	-	13.692	0.040	-	-
Tricyclo[2.2.1.0(2,6)]heptane, 1,7,7-trimethyl	508-32-7	14.417	0.113	-	-	-	-
Tridecanal	10486-19-8	27.387	0.010	26.119	0.010	26.746	0.010
Undecane	1120-21-4	-	-	-	-	18.599	0.010
α-Ionone	127-41-3	-	-	23.357	0.010	-	-
α-Phellandrene	99-83-2	12.543	1.677	-	-	-	-
α-Pinene	80-56-8	9.340	0.283	-	-	-	-

Table S2. EAG Responses of *A. mellifera* and *B. terrestris* to 14 synthetic compounds

**Compounds**	**0.1 μg/μL**		**1 μg/μL**		**10 μg/μL**		**100 μg/μL**	
	A. mellifera	*B. terrestris*	*A. mellifera*	*B. terrestris*	*A. mellifera*	*B. terrestris*	*A. mellifera*	*B. terrestris*
(*E,E*)-2,4-decadienal	15.75±2.41	26.86±2.94	36.68±2.22	73.74±6.8	144.69±6.49	195.02±14.06	203.43±7.92	327.52±11.36
(*E,E*)-2,4-hexadienal	5.81±0.83	9.55±1.67	31.57±2.69	39.51±2.77	152.67±4.04	149.19±6.75	349.34±8.91	394.53±7.14
(*E,Z*)-2,6-nonadienal	1.86±0.68	7.8±1.39	10.86±1.54	32.93±1.64	99.04±3.8	141.91±6.88	224.52±3.14	293.94±11
2-hexenal	29.25±4.05	7.85±1.56	48.76±5.13	22.43±1.12	116.78±9.26	86.72±5.49	416.99±9.31	354.16±18.82
hexanal	6.34±1.18	7.66±0.68	10.5±1.1	20.96±2.86	21.97±1.86	63.32±5.17	229.1±8.62	315.1±14.23
tridecanal	4.78±0.86	17.96±2.09	8.57±0.29	40.2±2.69	37.54±1.82	87.94±2.5	70.84±2.37	133.96±3.74
linalool	7.13±1.09	15.57±2.01	29.92±1.4	47.64±2.66	116.62±4.38	264.27±14.29	222.97±5.91	404.29±6.29
nerol	5.4±0.65	16.52±1.05	18.25±1.32	31.82±0.92	97.67±3.76	195.58±8.01	178.98±4.34	280.45±10.81
β-ionone	12.62±1.39	13.96±1.21	22.38±1.42	47.88±2.64	54.03±1.59	136.95±3.58	61.67±1.53	181.38±10.37
methyl salicylate	13.4±0.95	18.93±3.04	44.69±1.64	61.33±5.23	106.16±4.62	204.23±8.74	183.77±5.18	434.2±13.34
ethyl salicylate	10.23±1.51	11.8±1.43	31.22±3.26	47.04±2.18	98.8±3.16	148.94±4.5	178.22±8.16	263.44±7.62
hexanoic acid	5.48±1.27	4.88±1.92	24.16±1.76	15.17±2.07	159.06±6.39	66.36±1.65	533.08±13.68	174.77±5.69
phenylethyl alcohol	17.13±1.7	17.73±1.57	30.34±0.94	50.8±1.56	74.59±3.46	114.78±3.48	247.38±7.03	374.99±12.58
guaiacol	5.18±1.65	5.52±0.94	16.6±1.71	17.66±1.11	80.18±2.82	80.42±6.51	155.59±3.38	271.76±6.31

Table S3. Relationship between the intensity of the electroantennographic (EAG) responses and behavioral preferences of *Apis mellifera* and *Bombus terrestris* for six selected volatile compounds presented at different concentrations

ns: $P > 0.05$; *: $0.01 < P < 0.05$; **: $0.001 < P < 0.01$; ***: $P < 0.001$

Compounds	EAG				Behavioral preference			
	0.1 μg/μL	1 μg/μL	10 μg/μL	100 μg/μL	0.1 μg/μL	1 μg/μL	10 μg/μL	100 μg/μL
linalool	**	***	***	***	*	*	*	**
nerol	***	***	***	***	***	**	**	**
(*E*,*E*)-2,4-decadienal	*	**	*	***	*	***	***	**
2-hexenal	**	**	*	*	ns	***	*	ns
tridecanal	***	***	***	***	*	*	**	**
(*E*,*Z*)-2,6-nonadienal	**	***	**	**	ns	ns	**	*

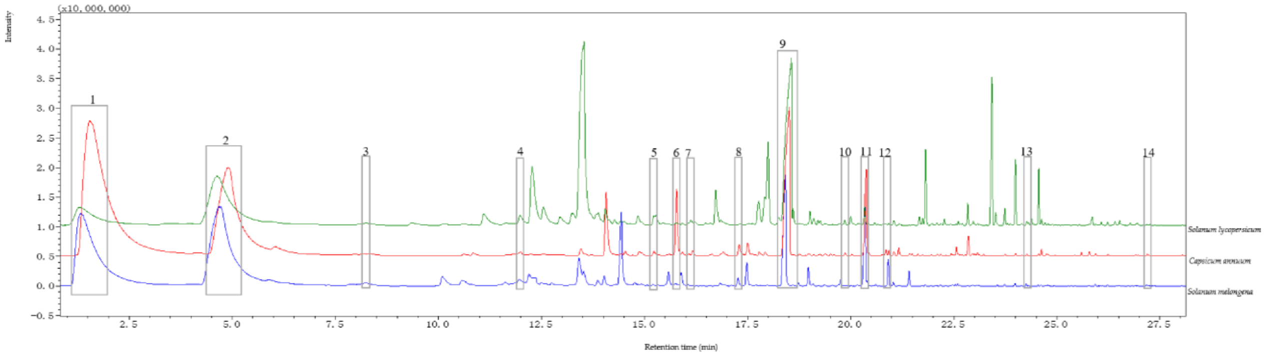

Figure S1. Total Ion Chromatogram (TIC) of Tomato, Pepper, and Eggplant Samples. 1: hexanal; 2: 2-hexenal; 3: (*E*,*E*)-2,4-hexadienal; 4: hexanoic acid; 5: guaiacol; 6: linalool; 7: phenylethyl Alcohol; 8: (*E*,*Z*)-2,6-nonadienal; 9: methyl salicylate; 10: nerol; 11: ethyl salicylate; 12: (*E*,*E*)-2,4-decadienal; 13: β-ionone; 14: tridecanal
